# Effect of Thermocycling and Surface Treatments on the Durability of Bond Strength of Translucent Zirconia to Resin Cement: An In Vitro Study

**DOI:** 10.7759/cureus.117251

**Published:** 2026-09-30

**Authors:** Bushra Alakeel, Mohammad Y. Hajeer, Issam Jamous

**Affiliations:** 1 Department of Fixed Prosthodontics, Faculty of Dentistry, Damascus University, Damascus, SYR; 2 Department of Orthodontics, Faculty of Dentistry, Damascus University, Damascus, SYR

**Keywords:** artificial aging, cojet system, resin cement, sandblasting, selective infiltration etching, shear bond strength, surface treatment, thermocycling, zirconia

## Abstract

Background: Achieving a durable bond between translucent zirconia and resin cement remains challenging due to the material's low surface energy and inherent smoothness. Surface treatment is essential for enhancing bond strength, and thermocycling is critical for predicting clinical performance.

Objective: This study aimed to investigate the influence of various substrate modifications on zirconia-resin bonding and to assess the impact of artificial aging on bond durability after 5,000 thermocycles.

Materials and methods: Seventy-two zirconia blocks were divided into three groups according to surface treatment: selective infiltration etching (SIE), sandblasting, and CoJet. Shear bond strength (SBS) was measured before and after thermocycling using a universal testing machine. SEM analysis was performed on the surface conditions, the debonded interface, and the failure patterns (adhesive, cohesive, or mixed). Data were analyzed using one-way ANOVA with Tukey's post hoc test and two-way ANOVA.

Results: Before thermocycling, the sandblasted and SIE groups showed higher SBS compared to the CoJet group. After thermocycling, the sandblasted group showed a 40% reduction in SBS, the CoJet group a 22% reduction, and the SIE group showed the least (9%). Adhesive failure was dominant in the sandblasted and CoJet groups, while the SIE group had more cohesive failures.

Conclusion: The SIE surface treatment demonstrated the highest bond strength and the greatest resistance to thermocycling, suggesting its potential as a reliable surface modification to enhance the durability of zirconia-resin bonding.

## Introduction

Over the past few decades, the use of zirconia in prosthodontics has significantly increased. It serves as a metal-free alternative to porcelain-fused-to-metal (PFM) restorations due to its mechanical strength, biocompatibility, favorable optical properties, and enhanced aesthetic appearance. Zirconia is commonly used in implant prosthodontics, specifically for single crowns and fixed partial dentures [1]. However, its use in the anterior region is limited due to its high opacity. This opacity is mainly attributed to the tetragonal crystalline phase, whose inherent photorefractive properties can cause light scattering [2]. Additionally, the presence of pores and impurities (such as alumina) [3] has necessitated the implementation of a "double-layer restoration" that includes a translucent ceramic layer. Monolithic translucent zirconia restorations may provide a solution to these challenges [4].

Since 2014, more translucent zirconia with an increased percentage of yttria (Y₂O₃) stabilizer has emerged in dentistry. This new zirconia features optimized translucency that can rival that of glass ceramics due to the introduction of the cubic zirconia phase. The cubic phase, with its homogeneous crystal structure, reduces light scattering at the grain boundaries, allowing light to pass through. Furthermore, changes in the zirconia structure, including reduced porosity and decreased additive amounts, may also contribute to improved translucency [4,5].

Establishing a strong, durable bond between yttria-stabilized tetragonal zirconia polycrystal (Y-TZP) and the veneer ceramic or resin cement has proven challenging [1,3]. This difficulty arises from the silica-free composition of zirconia, which makes it resistant to acid etching. As a result, etching with hydrofluoric acid, which is commonly used on silica-based ceramics, is ineffective on zirconia [6]. Consequently, multilayer zirconia structures are more prone to chipping and cracking during clinical use.

Various roughening techniques have been proposed to enhance adequate adhesion between resin cement and zirconia. The most widely used method is sandblasting with aluminum oxide (Al₂O₃) particles, which can vary in shape, size, and the duration and pressure of abrasion [7]. Sandblasting creates a finely textured surface on zirconia, which improves adhesive bonding by increasing surface energy, wettability, and surface area [8]. However, the stress from sandblasting may lead to cracking on the zirconia surface and can induce a phase transformation from tetragonal to monoclinic. Excessive treatment can result in the formation of transformed grains, a process that tends to reduce mechanical strength and impact the long-term performance of zirconia restorations [2,4].

Alternative techniques for conditioning the zirconia surface have been introduced, including pre-sintering sandblasting, selective leaching etching, friction chemical silica coating, plasma spraying, laser treatments, and silica coating. Silica coating creates a siliconized zirconia surface that can chemically bond with the applied saline, enhancing the adhesive bond to the resin [6]. Recent surface treatments have been proposed to improve the bond strength of zirconia and resin cement, such as selective infiltration etching (SIE). This procedure involves applying a hot glass infiltration agent to the zirconia, which creates eroded, porous surfaces, resulting in improved bond strength to the resin cement [9,10].

An additional crucial variable influencing the clinical outcomes for zirconia restorations is aging. Oral mechanical stresses, temperature, and humidity significantly influence bond degradation in zirconia restorations. Several studies have utilized thermocycling and varying water storage periods as artificial aging methods [11].

While previous studies have primarily focused on quantitative bond strength values, microstructural characterization of the treated surface and debonded surface morphology using scanning electron microscopy (SEM) is essential for understanding the adhesive interface. Thus, this study incorporated SEM and failure mode analysis to provide comprehensive insights beyond numerical data.

Therefore, this in vitro study aimed to evaluate the effects of various surface treatments and thermocycling (5,000 cycles) on the shear bond strength (SBS) of zirconia and to correlate these findings with SEM surface morphology and failure mode analysis to elucidate adhesion mechanisms and guide clinical selection. The null hypothesis was that neither the surface treatment nor aging would significantly affect the SBS.

## Materials and methods

Settings and sample size calculation

We conducted this comparative laboratory study in the Department of Fixed Prosthodontics, Faculty of Dental Medicine, Damascus University, from October 2024 to March 2025. The sample size was calculated using G*Power version 3.1.9 (Heinrich-Heine-Universität Düsseldorf, Düsseldorf, Germany). A sample size of 72 specimens was obtained based on the study by Lim et al. [12], using the following parameters: an effect size of f = 1.575, α = 0.05, power of 95%, and number of groups = 6.

Zirconia specimen preparation

The primary material used in this study was commercially available, high-translucency zirconia (DD cube X2; Dental Direkt, Spenge, Germany), which contains 5 mol% yttrium oxide (Y₂O₃) and is known as 5Y-TZP. Seventy-two cubic-shaped zirconia blocks were polished with silicon carbide papers using a gradual grit sequence: 600, 800, 1,000, and 1,200, using a polishing machine (Buehler, Lake Bluff, IL, USA) that is located in the Department of Physics, Faculty of Sciences, Damascus University, at 300 rpm, for surface standardization. Specimens were then placed in an ultrasonic bath for three minutes to remove any loose debris. To verify surface uniformity after polishing, surface roughness was measured for each specimen using a TR200 surface roughness tester. The mean initial surface roughness of all specimens was 0.2 µm, confirming surface homogeneity prior to treatment.

Methods for conditioning zirconia substrates

Group A

The specimens were sandblasted using 50 µm aluminum oxide powder (SHERA Werkstoff-Technologie GmbH, Lemforde, Germany), with the particles applied vertically to the zirconia substrate surface under a pressure of 0.25 MPa for 20 seconds at a working distance of 1 cm.

Group B

Tribochemical silica coating was performed using the CoJet™ system (3M ESPE, Saint Paul, MN, USA). Silica-modified aluminum oxide particles (CoJet Sand, 30 µm) were directed perpendicularly to the surface of the zirconia substrate under a pressure of 0.28 MPa for 20 seconds, while maintaining a constant working distance of 1 cm between the nozzle tip and specimen surface.

Group C

The specimens were treated based on the protocol described by Aboushelib et al. [9], using a laboratory-prepared paste from an infiltration glass powder composed of the following oxides: 65% SiO₂, 15% Al₂O₃, 10% Na₂O, 5% K₂O, and 5% TiO₂. This powder mixture was combined with distilled water to form the SIE mixture. Specifically, 0.035 g of this powder was mixed with 0.2 mL of deionized water to create a thin slurry. The slurry was carefully applied to the zirconia surface using a clean brush, evenly distributed using a vibrating technique, and air-dried for 60 seconds. The specimens were cooled to room temperature after thermal infiltration at 750°C for three minutes and then etched with 9.5% hydrofluoric acid for 20 minutes. Following surface treatment, all specimens were rinsed with distilled water, then cleaned in an ultrasonic water bath for three minutes, and air-dried before cementation. SEM (VEGA II, XMMU, TESCAN, Czech Republic) was performed after the surface treatments to further analyze the surface morphology.

Fabrication and bonding of composite resin cylinders to zirconia substrates

A total of 72 resin composite cylinders, each measuring 4 mm in diameter and 5 mm in height, were fabricated using a Plexiglas mold. The composite (Opallis Refill Composite; FGM, Joinville, Brazil) was packed into the mold, and 5-mm-thick Plexiglas slabs were placed on both the top and bottom surfaces to ensure flatness. Light curing was performed for 40 seconds from each side (top and bottom) using a light-curing unit with a wavelength of 480 nm. Subsequently, the composite cylinders were bonded to the treated zirconia surface using a self-adhesive resin cement (TheraCem, Bisco, Schaumburg, IL, USA) according to the manufacturer's instructions. To standardize the testing conditions and ensure a uniform cement thickness, a constant 5 kg load was applied to the samples using a pressure-standardizing apparatus at the Department of Fixed Prosthodontics, Damascus University. Excess cement was carefully removed with a curette and microbrush. Glycerin was applied around the bonding margin to prevent the formation of an oxygen-inhibition layer, followed by light curing for 40 seconds from two lateral sides and the top using the same curing device.

Aging

Following cementation, the samples were stored in distilled water at 37°C for 24 hours. After this period, half of the samples (n=36) were randomly divided into three subgroups (n=12 each) to investigate the initial bond strength. The remaining 36 samples were exposed to 5,000 thermocycles between 5°C and 55°C with a dwell time of 30 seconds and a transfer time of 10 seconds. After this, the SBS was measured.

SBS test

SBS testing for all samples (both initial and aged) was performed using a universal testing machine (Testometric, Rochdale, UK) at a crosshead speed of 1 mm/min with a 50 kgf load cell until bond failure occurred. The shear load was delivered via a chisel-edged blade, with the blade edge placed at the cement-zirconia interface and oriented parallel to the bonded surface. The SBS for each specimen was calculated by dividing the measured failure load (N) by the adhesive area (mm²). The failure loads were obtained during the debonding test. In this study, the bonding interface was circular, with a cylinder diameter of 4.0 mm, where the bonding area was 12.56 mm².

Failure mode analysis

After conducting SBS testing, fracture surfaces were examined using an SKT 41322 MEIJI TECHNO stereo microscope to determine the overall distribution of failure modes. Subsequently, the selection of specimens for SEM examination was based on the initial classification of failure modes observed under the stereo microscope. Two representative specimens were selected for each failure mode to illustrate the morphological characteristics of the failure surface. Since all specimens underwent uniform preparation and bonding conditions, morphological homogeneity was achieved across specimens, making the representative sample sufficient for qualitative morphological description. It is worth noting that SEM examination was intended for qualitative description rather than quantitative statistical analysis; therefore, examining all specimens was not necessary. Failure modes were classified into three types according to ISO 10365:2022 [13]: adhesive, cohesive, and mixed failure. To operationalize this classification, the Adhesive Remnant Index (ARI) was used, based on visual estimation of the amount of cement remaining on the zirconia surface after the shear bond test. Specimens were graded as follows: Score 0, no cement remaining on the zirconia surface or less than 25% remaining; Score 1, cement remaining between 25% and 75%; and Score 2, cement remaining more than 75%. Failure was considered adhesive when the score was 0, mixed when the score was 1, and cohesive when the score was 2. Assessment was performed using a stereo microscope at 40× magnification.

Statistical analysis

All statistical analyses were performed using IBM SPSS Statistics for Windows, Version 28.0 (Released 2021; IBM Corp., Armonk, NY, USA). The normality of the data distribution was assessed using the one-sample Kolmogorov-Smirnov test. To compare the mean SBS values among the surface treatment groups before thermocycling (24-hour water storage), a one-way ANOVA was performed, followed by Tukey's post hoc test for multiple pairwise comparisons. To evaluate the effect of thermocycling on bond strength within each group as well as the interaction between surface treatment and aging condition, a two-way ANOVA was employed. The significance level was set at 5%.

## Results

Initial SBS (before thermocycling)

The mean SBS values for the three surface treatment groups before thermocycling are presented in Table 1 and illustrated in Figure 1. Among all groups, the SIE group exhibited the highest mean SBS (21.33 ± 0.42 MPa), followed by the sandblasted group (13.29 ± 0.63 MPa), whereas the CoJet group showed the lowest mean SBS (8.90 ± 0.48 MPa).

**Table 1:** Mean values of SBS and SD for each study before and after thermocycling, along with the reduction ratio (%). SBS: shear bond strength, SD: standard deviation; SIE: selective infiltration etching.

Group	Mean SBS before thermocycling (in MPa ± SD)	Mean SBS after 5,000 thermocycles	Reduction ratio
Sandblasted	13.29 ± 0.63	7.33 ± 0.29	40%
CoJet	8.90 ± 0.48	6.89 ± 0.22	22%
SIE	21.33 ± 0.42	19.36 ± 0.40	9%

**Figure 1:**
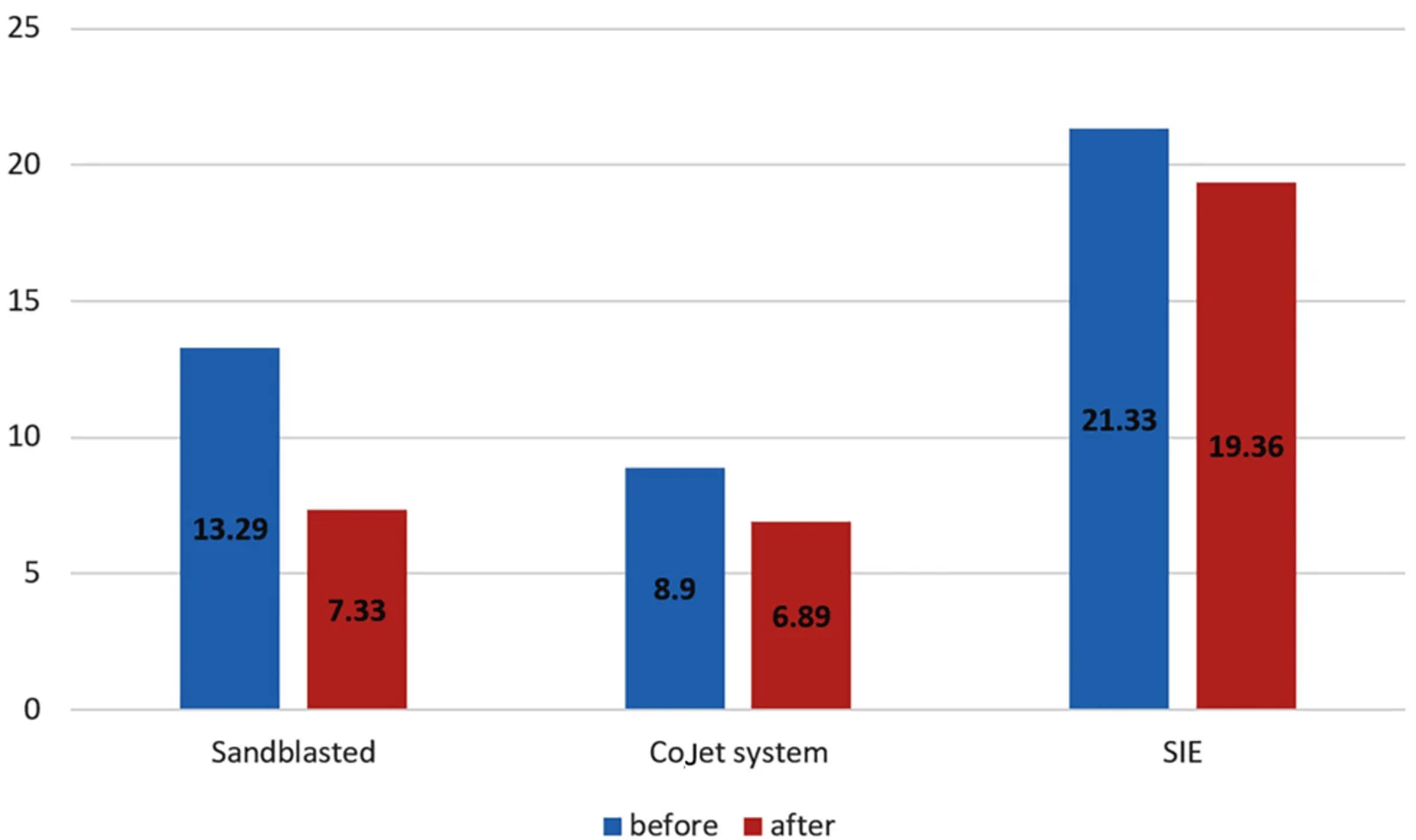
Bar chart showing the mean SBS of the three study groups before and after thermocycling. SBS: shear bond strength; SIE: selective infiltration etching.

The results demonstrate a significant effect of surface treatments on bond strength. One-way ANOVA revealed statistically significant differences among the groups after 24 hours of water storage (F = 1788.98, p < 0.001). To determine statistically significant differences in the average SBS between the study groups, Tukey's post hoc multiple comparisons test was performed, and the results are shown in Table 2.

**Table 2:** Tukey's post hoc comparisons of mean SBS differences between the study groups before thermocycling (initial bond strength). SBS: shear bond strength. ^*^Statistically significant.

Group (I)	Group (J)	Mean difference (I-J)	Standard error	Probability value
Sandblasted	CoJet	4.389	0.211	<0.001*
	SIE	-8.044	0.211	<0.001*
CoJet	SIE	-12.433	0.211	<0.001*

Effect of thermocycling on SBS

After exposure to 5,000 thermocycles (5°C-55°C), the SBS values decreased in all groups (Table 1, Figure 1). The highest mean SBS after aging was observed in the SIE group (19.36 ± 0.40 MPa), followed by the sandblasted group (7.33 ± 0.29 MPa), while the CoJet group continued to show the lowest values (6.89 ± 0.22 MPa).

All study groups that underwent surface treatment experienced significant reductions in bond strength upon thermocycling. Notably, the sandblasted group was the most vulnerable to aging, experiencing a sharp 40% decline. In contrast, the CoJet group showed a considerable decline in bond strength, with a 22% reduction, resulting in the lowest post-aging SBS, while the SIE group demonstrated remarkable stability, with a minimal 9% reduction (Table 1).

A two-way ANOVA was conducted to evaluate the effects of surface treatment, thermocycling, and their interaction on SBS. The analysis revealed a statistically significant main effect of surface treatment on SBS (F = 4221.460, p < 0.001), indicating that surface treatment type significantly influenced bond strength. Additionally, a significant main effect of thermocycling was observed (F = 797.491, p < 0.001), confirming that thermocycling substantially reduced SBS values. Furthermore, a statistically significant interaction effect was found between surface treatment and thermocycling (F = 127.158, p < 0.001), indicating that the effect of aging on bond strength varied by surface treatment (Table 3).

**Table 3:** Two-way ANOVA test results for the effects of surface treatment, thermocycling, and their interaction on SBS. SBS: shear bond strength; df: degree of freedom.

Source	df	F	p-value	Partial η²
Group	2	4221.460	<0.001	0.992
Thermal rounds	1	797.491	<0.001	0.924
Interaction (group * thermal rounds)	2	127.158	<0.001	0.794

Failure mode distribution

The distribution of failure modes for all groups before and after thermocycling is presented in Figure 2. Before thermocycling, adhesive failure dominated the sandblasted (58.3%) and CoJet (50%) groups, followed by mixed (33.3% for both) and cohesive (8.3% and 16.7%, respectively) modes. In contrast, the SIE group demonstrated remarkable superiority, with cohesive failure being the predominant pattern at the highest rate (83.3%), followed by mixed failure at 16.7%, and a complete absence of adhesive failure, confirming the superior strength of the adhesive interface in this group.

**Figure 2:**
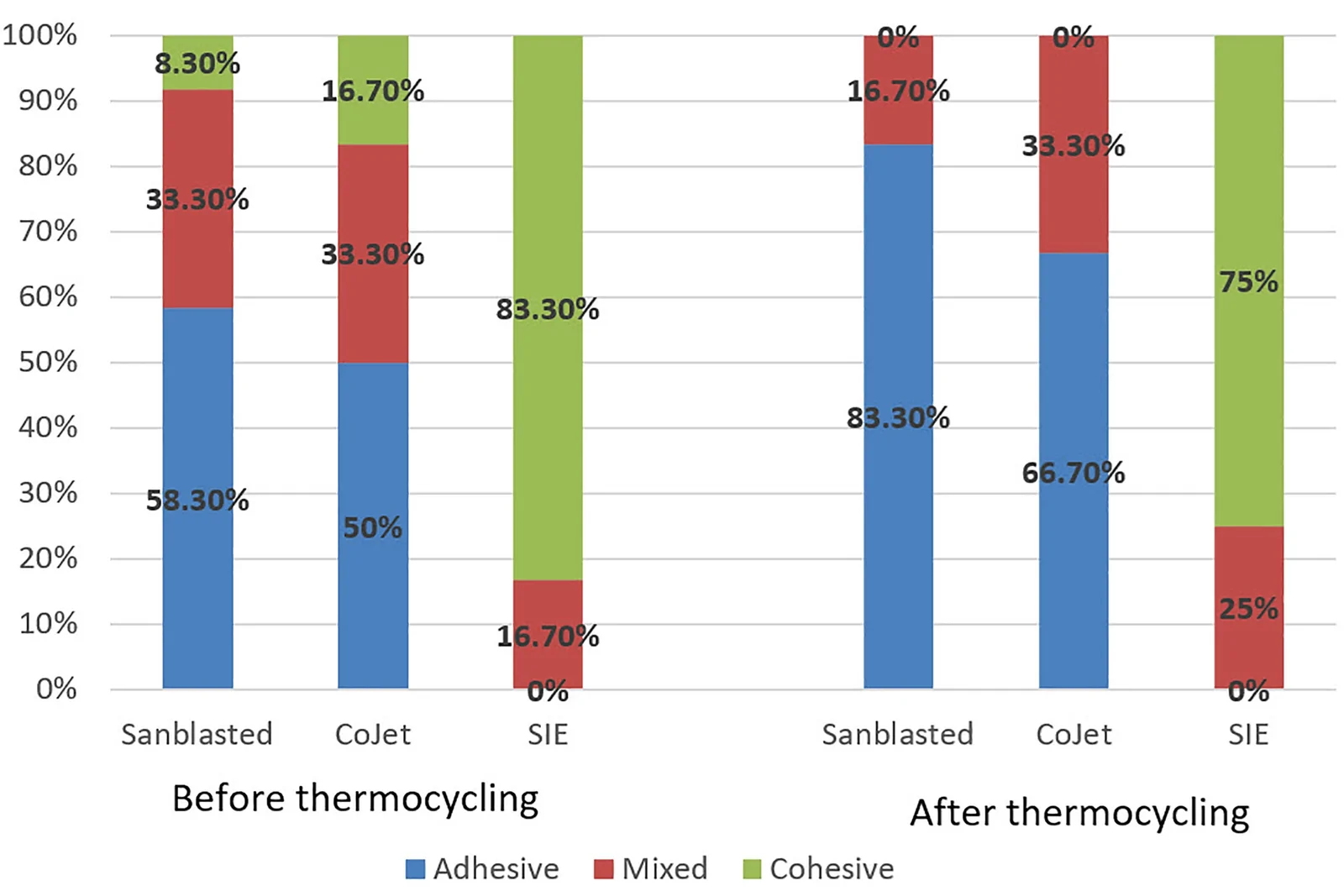
Bar chart showing the percentage distribution of failure patterns at the adhesion surface before and after exposure to thermal cycles, according to the study group. SIE: selective infiltration etching.

After thermocycling, cohesive failure was completely absent in both the sandblasted and CoJet groups. The sandblasted group maintained adhesive predominance (83.3%), with mixed failure declining to 16.7%. The CoJet group showed an increase, with adhesive failure rising to 66.7%. In contrast, the SIE group sustained its clear superiority, with cohesive failure remaining the most prevalent mode (75%), followed by mixed failure (25%), and a persistent complete absence of adhesive failure, reflecting the durability and stability of this surface treatment even after exposure to thermal stresses.

Surface morphology analysis

Figure 3 presents characteristic SEM micrographs of zirconia surfaces after undergoing different surface treatments at various magnifications. In Figure 3A, the sandblasted specimen reveals an irregular topography characterized by deep grooves, a pitted morphology with sharp edges, and angular peaks formed by the impact of alumina particles. While this surface roughening increases the surface area available for micromechanical interlocking, it also creates localized microcracks and stress concentrations that may compromise structural cohesion. In contrast, the surface treated with CoJet shows isolated clusters of silica-rich nodules and "islands" adhered to the zirconia support (Figure 3B). These surface remnants, consisting of silica-coated alumina particles, exhibit moderate reactivity and increased roughness, which can facilitate silane coupling, although their distribution across the surface is not uniform. The specimens treated with SIE before etching exhibit a dense, glazed surface composed of molten, spherical glassy beads (Figure 3C). This formation results from the thermal infiltration of a silica-alumina-alkali glass layer into the grain boundaries, creating a chemically modified surface that is dense and free of mechanical defects. Upon etching with hydrofluoric acid, the SIE-treated specimens reveal a distinct honeycomb-like micro- and nanoporous structure (Figure 3D). This structure arises from the selective dissolution of the infiltrated glassy phase, resulting in an interconnected network of pores and channels. This topography significantly enhances micromechanical retention and resin infiltration, thereby strengthening the adhesion between the resin cement and zirconia.

**Figure 3:**
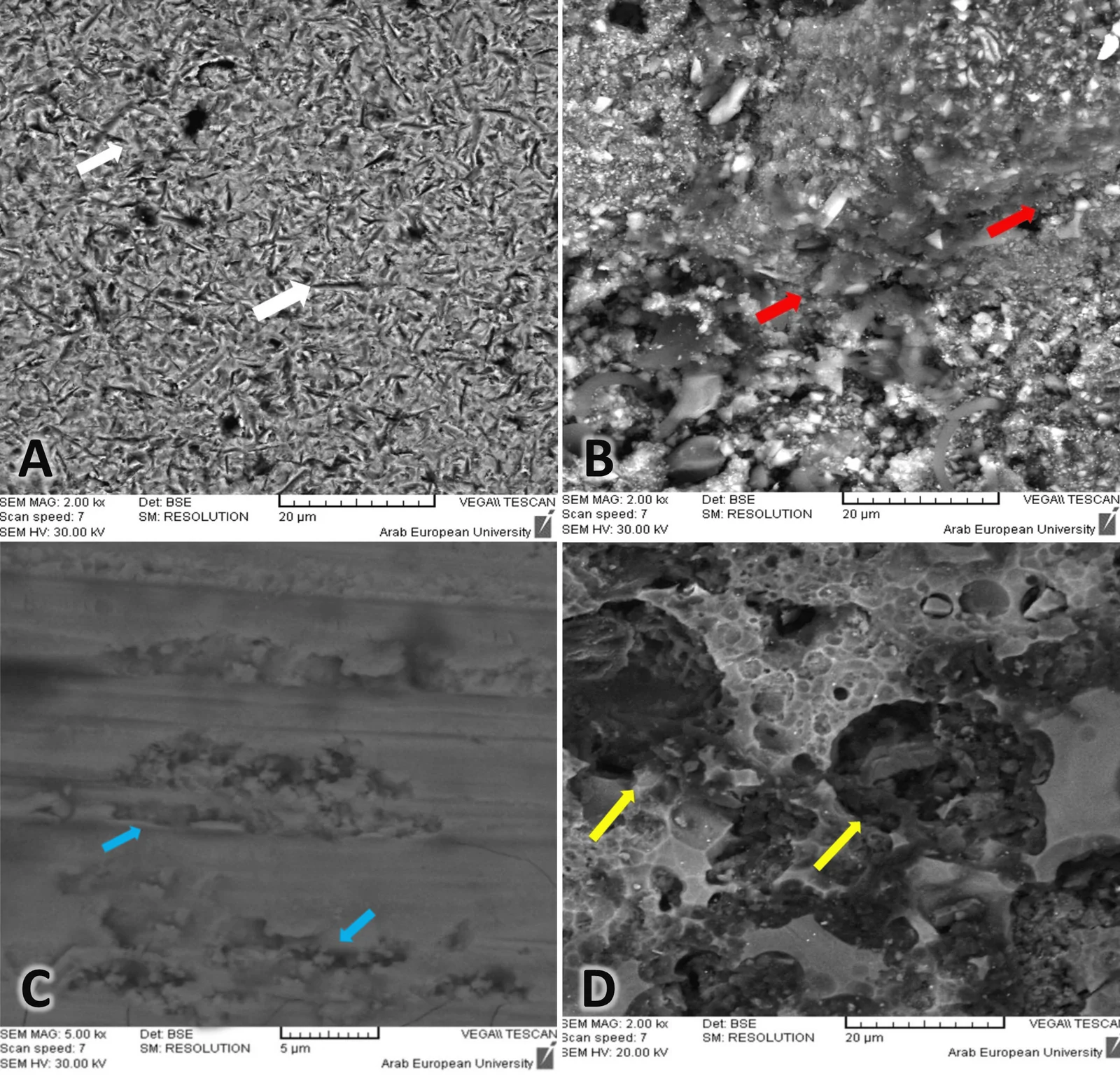
Representative SEM images showing morphological changes of the treated zirconia surfaces. (A) Sandblasted, (B) CoJet, (C) SIE, and (D) SIE after etching. The white arrows indicate sharp edges in group A, while the red arrows in group B point to clusters of silica islands, the blue arrows in C indicate fused round beads, and the yellow arrows in D highlight the honeycomb-like due to the etching. SEM: scanning electron microscopy; SIE: selective infiltration etching.

SEM analysis of fractured specimens showed distinct failure modes among the experimental groups. The adhesive failure pattern was prevalent in both the sandblasted and CoJet groups, where the debonded zirconia surfaces appeared clean with a small amount of resin cement, indicating weak interfacial bonding. In contrast, the specimens treated with SIE predominantly exhibited cohesive failures, with fractures occurring within the resin cement and the zirconia surfaces fully covered by resin residues, suggesting a strong micromechanical and chemical bond. Additionally, some mixed failures were noted, characterized by partial resin residues, reflecting intermediate adhesion strength between the two extremes. Consistent with the numerical data, the SIE surface treatment exhibited the most stable and durable bonding behavior, with minimal degradation after thermocycling, as illustrated in Figure 4.

**Figure 4:**
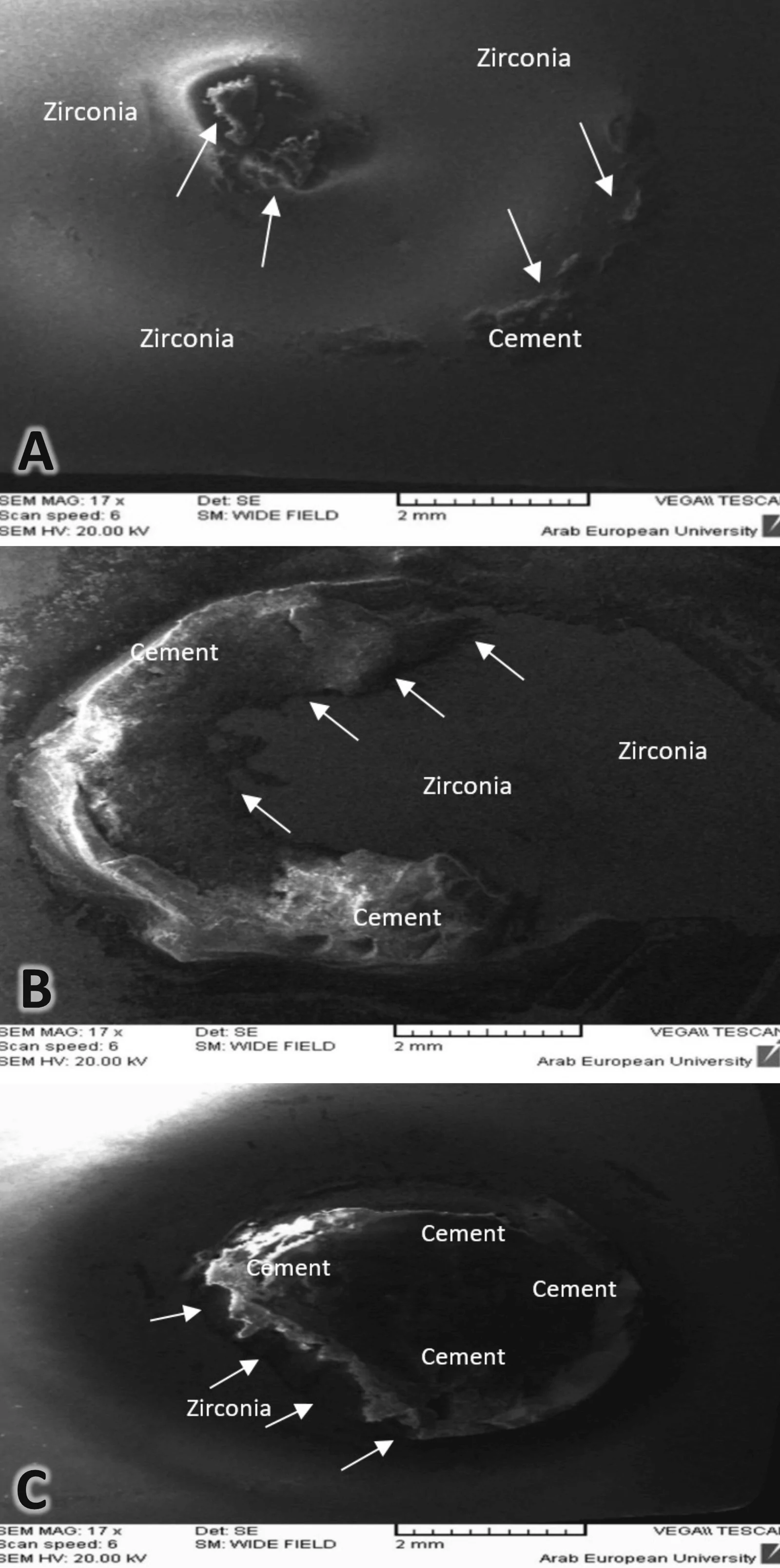
Representative SEM images displaying debonding patterns at 17× magnification. (A) Interfacial failure (<25% cement remnants), (B) mixed failure (50% retained), and (C) cohesive failure (75% cement with composite). White arrows indicate the demarcation boundaries between zirconia and residual cement in all micrographs. SEM: scanning electron microscopy.

## Discussion

Translucent zirconia is widely used in dental restorations due to its superior aesthetic properties and mechanical strength [12]. However, achieving a durable bond between zirconia and resin cement is challenging because of the material's low surface energy and inherent smoothness, which limit its bondability [10]. Consequently, surface treatment of zirconia is crucial for enhancing bond strength to resin cements [14].

Surface treatments can generally be classified into two categories: chemical and mechanical. Chemical treatments often involve the application of adhesion agents, such as methacrylate-based primers, which improve the chemical interaction between the zirconia surface and the resin cement. Mechanical treatments, such as sandblasting or laser irradiation, increase the surface roughness of zirconia, thereby improving the retention of resin cements [1,8]. Additionally, thermal changes significantly impact the long-term durability of zirconia restorations. Thermocycling simulates the thermal fluctuations that occur in the oral environment due to hot and cold food or beverages [15]. Repeated cycles of heating and cooling can create thermal stresses that affect both the bond strength and long-term stability of zirconia-to-resin bonds [16]. The effect of thermocycling on bond durability depends strongly on the surface treatment and cement used, as some treatments can withstand thermal cycling better than others [6].

This research highlights the significance of surface modification and the necessity of thermocycling simulation for predicting the clinical performance of zirconia restorations. Understanding the interaction between these factors is essential for enhancing the longevity and reliability of zirconia-based dental restorations. According to the findings of this study, the first null hypothesis was rejected.

After 24 hours of water storage, the SIE group demonstrated superior bond strength compared to all other groups, while the CoJet group exhibited lower values than the conventional sandblasted group. The high SBS values of the SIE group are directly attributed to the formation of a three-dimensional porous network, which enables nanomechanical interlocking with the resin cement. SEM analysis confirmed resin infiltration within the inter-grain porosity created by the heat-induced maturation (HIM)/SIE surface treatment, leading to enhanced micromechanical retention. In contrast, the CoJet group demonstrated significantly lower SBS values compared with the sandblasted group. This limited performance is attributed to its reliance on a tribochemical silica coating, which, in this study, failed to achieve complete silica coverage [17], where uncovered areas present a non-cohesive silica layer acting as a physical barrier that prevents direct cement-zirconia contact. Additionally, aluminum residues may remain after tribochemical treatment, interfering with MDP-zirconia bonding [18]. Additionally, the high hardness of zirconia may limit adequate silica impregnation during the tribochemical silica coating [19]. In contrast, sandblasting achieved higher bond strength than the CoJet group by relying on micromechanical interlocking through alumina-induced deep roughness and retentive pits, a mechanism that appeared more effective under the current experimental conditions. Conversely, CoJet depends on a tribochemical silica coating. Our SEM findings support this, revealing non-fused silica aggregates on CoJet surfaces, whereas sandblasted samples exhibited deep, uniform roughness with distinct retentive pits.

After thermocycling, all tested groups exhibited a notable bond strength reduction, indicating that artificial aging factors adversely affected bond integrity. Consequently, the second null hypothesis was rejected.

The decline in bond strength after thermocycling varied considerably among groups: 9% for SIE, 22% for CoJet, and 40% for the sandblasted group. The SIE group exhibited the least reduction due to its three-dimensional porous network, which provides nanomechanical intrusion and distributes thermal stress evenly. Recent studies confirm that surfaces with controlled porosity offer better aging resistance compared to purely rough or purely chemically active surfaces [18,20]. Thus, SIE's 9% reduction reflects an optimal stability between mechanical retention and chemical bonding, yielding the most durable bond under thermal aging conditions. In contrast, the 22% reduction in the CoJet group reflects incomplete silica coverage and residual alumina interference. Recent systematic reviews confirm that silica coating alone without silane does not provide reliable long-term adhesion to zirconia [20]. In contrast, the 40% reduction in the sandblasted group is attributed to degradation of the resin cement's mechanical properties during water storage [21]. Furthermore, the presence of sharp edges and microcracks also contributes to stress concentration zones [22] and the lower hydrolytic stability of Z-O-P bonds of the cement [23,24].

The present findings of this study align with previous studies [9,10], reporting that the SIE technique achieved the highest bond strength before and after thermocycling. However, some authors reported a decrease in SIE bond strength after thermocycling, observing a 58.5% reduction [25]. This discrepancy is mainly attributed to differences in the type of glass powder used (Cercon Ceram Kiss; Dentsply Sirona, Hanau, Germany), the aging protocol (21 days of water storage followed by 6000 thermocycles), and the resin cement employed (Panavia F2.0; Kuraray Noritake Dental, Tokyo, Japan). Conversely, the results of this study differed from those of Samimi et al. [26], who found that the SIE method did not significantly improve bond strength compared to sandblasting. This discrepancy may be explained by several methodological factors, including their use of a microshear bond strength test with a small bonding area (1 mm²), which renders specimens more susceptible to premature failure due to non-uniform stress distribution and rapid water penetration that accelerates hydrolytic degradation. Additionally, the high sandblasting pressure (3.5 bar) employed in their study provides a sandblasted surface with high initial bond strength.

Furthermore, the results of this study support the findings reported by Hayran et al. [21], which showed that surface treatment of zirconia with the CoJet system reduces adhesion to resin cement. In contrast, sandblasting treatment enhanced the adhesive bond strength between zirconia and resin cement. However, the outcomes of this study differ from those reported in some studies [27], which indicated that CoJet application enhanced the surface energy and promoted durable, long-term bonding compared to sandblasting. This discrepancy may be attributed to variations in the types of zirconia used, which can consequently alter adhesive behavior [28,29]. Overall, the strength of correlation reported across studies cannot be directly compared because of varying study conditions, including testing methods, bonding surface areas, and aging protocols.

Thermal treatment and exposure to environmental situations, such as moisture or heat, are critical factors that can influence failure patterns, highlighting the need for innovative strategies to mitigate these effects. Adhesion failure was notably prevalent in the sandblasted and CoJet groups, especially after thermocycling. This may be attributed to thermocycling exposing a larger surface area of the bonded interface to the hydrolytic effects, thereby creating additional pathways for water penetration into the interface [9]. Furthermore, as monoclinic phase transformation progresses from the zirconia surface toward the bulk, microcracks and tensile residual stresses may develop, potentially reducing flexural strength [15,30].

SEM images in Figure 5 demonstrated clear morphological differences in the zirconia-resin interfaces across the various surface treatment groups. The sandblasted and CoJet groups exhibited minimal surface roughness and poor resin infiltration, resulting in limited mechanical interlocking. In contrast, the SIE-treated samples displayed significant micro- and nanoscale porosities with resin infiltration, enhancing micromechanical interlocking and improving adhesive performance.

**Figure 5:**
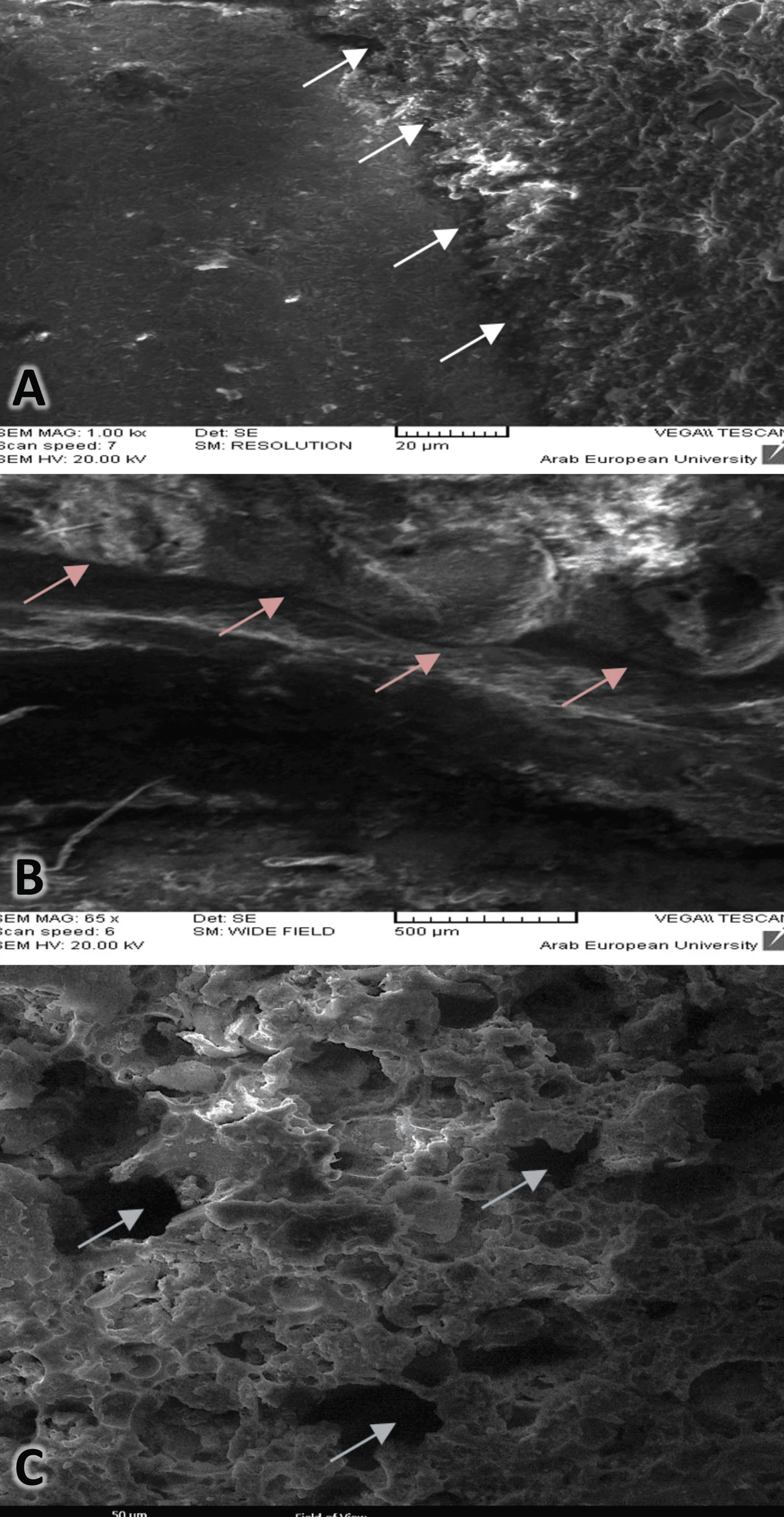
Representative SEM images showing zirconia-resin interface. Groups conditioned with 50 µm particles (A) and CoJet (B) exhibited a lack of surface irregularities with minimal resin retention, as indicated by the white arrows in (A) and the pink arrows in (B). In contrast, the SIE-treated group (C) demonstrated deep resin infiltration into the three-dimensional nanopores, highlighted by the gray arrows.

Study limitations

This study has several limitations that should be considered when interpreting the findings. First, only one type of zirconia (5Y-TZP) and one resin cement (TheraCem) were evaluated, restricting generalizability. Second, bond strength was tested without mechanical loading or masticatory simulation, and laboratory conditions may not reflect clinical variability. Third, failure mode analysis was limited to qualitative SEM observations and did not include quantitative image analysis. Finally, the effect of surface treatment on the phase transformation or flexural strength of zirconia, pH fluctuations, and enzymatic challenges were not investigated. Future studies should address these aspects.

## Conclusions

Within study limits, the SIE surface treatment outperformed the other groups, with the highest shear bond strength before and after thermocycling with only a 9% reduction, owing to its three-dimensional porous network that enhances nanomechanical interlocking and thermal stress distribution. The CoJet group, however, exhibited limited bond strength with a 22% reduction, which is explained by incomplete silica coverage and the presence of unfused silica aggregates acting as a physical barrier. As for the sandblasted group, although it started with moderate initial bond strength, it demonstrated the greatest reduction with 40% after thermocycling, likely due to stress concentration zones from microcracks and the lower hydrolytic stability of Zr-O-P bonds. Therefore, future studies mimicking oral environments are warranted to confirm these findings and further develop the clinical SIE technique.
